# Evaluation of patient satisfaction of an outpatient gastroscopy service in an Asian tertiary care hospital

**DOI:** 10.1186/1471-230X-12-96

**Published:** 2012-07-28

**Authors:** Najib Azmi, Wah-Kheong Chan, Khean-Lee Goh

**Affiliations:** 1 Division of Gastroenterology, Department of Medicine, Faculty of Medicine, University of Malaya, 50603, Kuala Lumpur, Malaysia

**Keywords:** Gastroscopy, Patient satisfaction, Patient comfort, Waiting time

## Abstract

**Background:**

There are limited published studies on patient satisfaction towards endoscopy from Asian countries. Different methods of evaluation of patient satisfaction may yield different results and there is currently no study to compare results of on-site versus phone-back interviews.

**Method:**

On-site followed by phone-back interviews were carried out on consecutive patients attending the outpatient gastroscopy service of University of Malaya Medical Centre between July 2010 and January 2011 using the modified Group Health Association of America-9 (mGHAA-9) questionnaire. The question on technical skill of endoscopist was replaced with a question on patient comfort during endoscopy.

**Results:**

Seven hundred patients were interviewed. Waiting times for appointment and on gastroscopy day, and discomfort during procedure accounted for over 90% of unfavorable responses. Favorable response diminished to undesirable level when waiting times for appointment and on gastroscopy day exceeded 1 month and 1 hour, respectively. Satisfaction scores were higher for waiting time for appointment but lower for personal manner of nurses/staff and explanation given during phone-back interview. There was no significant difference in satisfaction scores for other questions, including overall rating between the two methods.

**Conclusion:**

Waiting times and discomfort during procedure were main causes for patient dissatisfaction. Phone-back interview may result in different scores for some items compared with on-site interview and should be taken into account when comparing results using the different methods.

## Background

Patient satisfaction is considered a measure of a high-quality endoscopy [1], and many endoscopy units administer patient satisfaction surveys for quality-control purposes [2]. Deficiencies in an endoscopy unit can be identified through such studies and these can then be analyzed and solutions can be made to improve the overall quality of endoscopy. Patient satisfaction also affects health care outcomes. Patients who are dissatisfied are likely to be non-compliant [3], transfer care to other centres [4] and engage in litigation issues [5]. From the business point of view, satisfaction scores can be used for marketing purposes if they are sufficiently impressive [6].

The Endoscopy Suite in University of Malaya Medical Centre caters to 3000 – 3200 gastroscopies per year. In the year 2010, over 2000 outpatient gastroscopies were performed and the number is increasing. Despite this figure, no studies have been done to assess patient satisfaction. Therefore we decided to perform a survey to assess patient satisfaction of our outpatient gastroscopy service and to identify areas of dissatisfaction for improvement. Moreover, there are limited published studies on patient satisfaction towards endoscopy from Asian countries.

Many studies on patient satisfaction were carried out immediately after the procedure [7-9]. Sedation given during the procedure may affect patient satisfaction score and this raises the question on whether answering a questionnaire immediately after the procedure may yield different satisfaction scores compared to administering the questionnaire at a later date. In a study by Lin et al [10], on-site survey resulted in higher satisfaction scores compared to mail back survey. Besides the possible influence of sedation, it was hypothesized that patients may feel disinclined to give low satisfaction scores in the presence of endoscopy unit staff. Another study by Harewood et al [11] reported that survey methods that involved more personal interaction such as on-site surveys and phone interviews tend to generate higher response rates than less personal methods such as mail back surveys and electronic mail surveys. To the best of our knowledge, there is till date no study comparing on-site interview and phone interview in terms of success rate and patient satisfaction score of endoscopy services. Thus, the secondary aim of our study is to compare the results of immediate on-site interview and delayed phone interview in these aspects.

### Outpatient gastroscopy Service in University of Malaya Medical Centre

Our center practices an open-access outpatient gastroscopy service receiving patients from primary care clinics, other specialist clinics and those discharged from in-patient wards in addition to patients from the gastroenterology clinic. Gastroscopy appointments are given on a first-come-first-serve basis. When a patient is deemed to require an earlier gastroscopy appointment, the doctor-in-charge would negotiate the patient’s appointment to an earlier date. Appointment time on gastroscopy day is staggered fifteen minutes per patient per room to reduce waiting time. A support staff will register patients and a staff nurse will help patients prepare for the procedure. Explanation about the procedure is given and consent is obtained by the endoscopist before the procedure. Two rooms run simultaneously for gastroscopy during each session. Blood pressure, pulse rate, respiratory rate and oxygen saturation is recorded before the procedure. Lignocaine 1% pharyngeal spray is administered to all patients. All patients receive intravenous Midazolam 2.5 mg to 5 mg as sedation prior to the procedure unless they have requested not to be given sedation or it is deemed unsafe by the endoscopist. The dosage is given at the discretion of the endoscopist based on subjective assessment. Gastroscopy is performed by various grades of endoscopist including consultants, specialists, and trainees under supervision. Different types of gastroscopes with varying diameters are used. During the procedure, pulse rate and oxygen saturation is monitored continuously. Following the procedure, patients rest in the recovery area till they regain full consciousness before they are seen by the endoscopist-in-charge at the discharge counter who would explain the gastroscopy findings to them before they go home.

## Methods

This is a cross sectional study of consecutive patients attending the outpatient gastroscopy service in University of Malaya Medical Centre between July 2010 and January 2011. Written informed consent was obtained from all patients and the study was approved by the ethical committee of this institution.

### During gastroscopy

The observer assessment for alertness/sedation scale (OAASS) [12] was used in this study as an objective measurement of the level of patient sedation just before the procedure began (OAASS scale: 1 – 5). The scale is sensitive to the amount of midazolam administered [12] and correlates well with the American Society of Anesthesiology (ASA) level of sedation [13] (ASA level of sedation: mild, moderate, deep correlates with OAASS scale: 5, 2 – 4, 1, respectively).

### On-site interview

The interview was carried out using an investigator-administered questionnaire (see below) in an isolated room in the Endoscopy Suite immediately after the patients have received explanation from and were discharged by their endoscopist. Additional information such as name, telephone number, age, sex, race, education level, previous gastroscopy, waiting time for appointment, waiting time on gastroscopy day, indication for gastroscopy, duration of the procedure and sedation given were recorded. Waiting time for appointment refers to the duration from the day the gastroscopy was planned to the day that it was performed and was categorized as < 1 week, 1 – 2 weeks, 2 – 4 weeks and > 4 weeks. Waiting time on gastroscopy day refers to the duration from the time of registration on the day of the procedure to the time the procedure was performed and was categorized as < ½ hour, ½ - 1 hour, 1 – 2 hours and > 2 hours.

### The questionnaire and assessment of patient response

We used the modified Group Health Association of America-9 (mGHAA-9) questionnaire but replaced the question on technical skill of endoscopist with a question on patient comfort level during endoscopy as proposed by Rio et al [14]. The questionnaire consists of the following: Q1 – Length of time spent waiting for the appointment, Q2 – Length of time spent waiting at the Endoscopy Suite for the procedure, Q3 – Personal manner of the physician who performed the procedure, Q4 – Personal manner of the nurses and other support staff, Q5 – Adequacy of explanation of what was done for you, Q6 – Comfort level during the procedure, Q7 – Overall rating of the visit, Q8 – Would you have the procedure done again by this physician? Q9 – Would you have the procedure done again at this facility? The original ordinal five-value Likert scale (excellent, very good, good, fair, and poor) was used. Patient response for each of the questions Q1 to Q7 was dichotomized to favorable (excellent, very good, good) and unfavorable (fair, poor). The percentages of favorable and unfavorable responses for each of the questions were calculated. A problem rate was also estimated by dividing the sum of unfavorable responses with the sum of questions asked and multiplying by 100. A Pareto chart was used to illustrate the contribution of each of the questions to the overall unfavorable responses. Finally, the percentages of favorable and unfavorable responses were estimated across the categories of waiting time for appointment and waiting time on gastroscopy day.

### Telephone interview

All patients were contacted by phone within a month from the day of the procedure for a second interview using the same questionnaire. Patients who did not respond after 3 random calls were excluded. The interval of the phone-back interview from the day of the procedure was recorded. A different interviewer not involved in the on-site interview and who was blinded to the response of the on-site interview administered the questionnaire through phone interview. Patients who were unwilling to participate or did not answer all the questions were excluded. We gave a score to patient response for each of the questions Q1 to Q7 (poor = 1, fair = 2, good = 3, very good = 4, excellent = 5) to compare patient response during on-site interview and during phone-back interview.

### Statistical analysis

Data were analyzed using a standard statistical software program (SPSS 16.0). Continuous variables were expressed as means with standard deviations. Categorical variables were analyzed using chi-square test. Variables with p-value < 0.20 on univariate analysis were entered into multivariate analysis using logistic regression. Mean and median scores for each of the questions Q1 to Q7 as well as the mean and median total scores for on-site interview and for phone-back interview were calculated. The median scores for each of the questions for the two groups were compared using Wilcoxon Signed Rank Sum test. Significance was defined as p-value < 0.05.

## Results

A total of 735 patients came for outpatient gastroscopy during the study period. Seven hundred patients were interviewed. Twenty eight patients declined to participate while seven others were excluded because of considerable language barrier. Mean age of the study population was 54.9 ± 15 years, with minimum age of 15 years old and maximum age of 91 years old. Patient characteristics and procedure-related information are shown in Table 1.

**Table 1:** Patient characteristics and procedure-related information

	
Gender, n (%)	
Male	326 (47%)
Female	374 (53%)
Race, n (%)	
Malay	203 (29%)
Chinese	290 (41%)
Indian	199 (28%)
Others	8 (1%)
Education level, n (%)	
None	106 (15%)
Primary	115 (16%)
Secondary	299 (43%)
Tertiary	180 (26%)
History of previous gastroscopy, n (%)	
Yes	299 (43%)
No	401 (57%)
Indication, n (%)	
Suspected peptic ulcer disease	454 (65%)
Suspected malignancy	100 (14%)
Gastroesophageal reflux disease	73 (10%)
Variceal surveillance	21 (3%)
Anemia for investigation	47 (7%)
Procedural	5 (1%)
Duration of gastroscopy, n (%)	
≤ 10 minutes	407 (58.1%)
> 10 minutes	293 (41.9%)
Sedation, n (%)	
Yes	641 (92%)
No	59 (8%)
Amount of midazolam given, n (%)	
≤ 2.5 mg	238 (37.1%)
> 2.5 mg	403 (62.9%)
Level of sedation according to OAASS, n (%)	
1	2 (0.3%)
2	60 (8.6%)
3	426 (60.9%)
4	110 (15.7%)
5	102 (14.6%)

The two patients who were deeply sedated (OAASS = 1) were grouped into the same group as patients who were moderately sedated (OAASS = 2 – 4) due to the small number and for ease of statistical analysis.

OAASS = observer assessment for alertness/sedation scale.

### Patient response for Q1 to Q6

**Figure 1:**
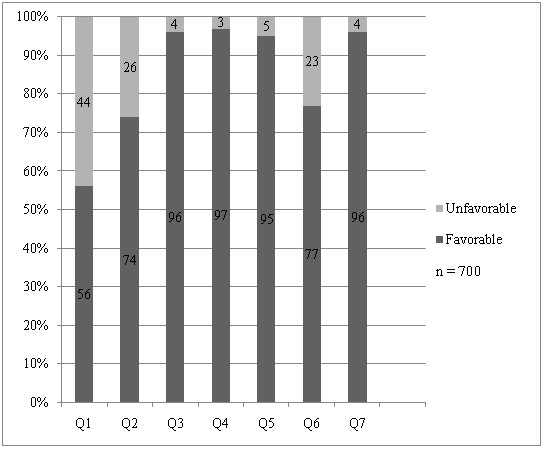
Patient responses for questions Q1 to Q7.

The questions which had the most unfavorable responses were that on waiting time for appointment followed by waiting time on gastroscopy day and comfort level during procedure. High favorable response rates were seen for the other 3 questions (Figure 1).

### Problem rate and pareto analysis

**Figure 2:**
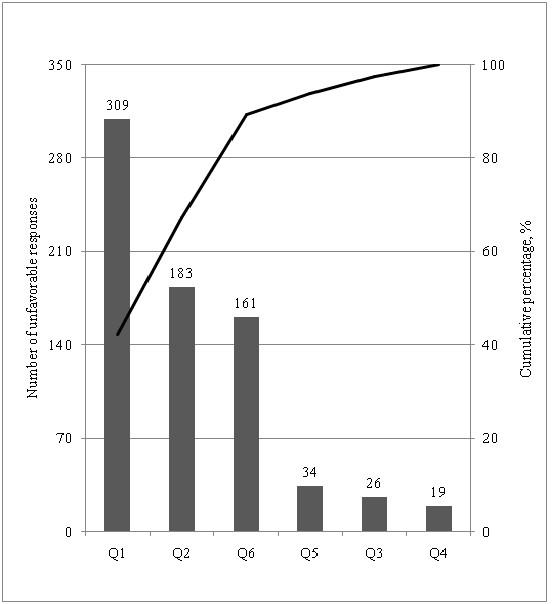
**Pareto chart showing the contribution of each of the questions to unfavorable responses.** The bars represent the number of unfavorable responses for each of the questions Q1 to Q6 (total number of unfavorable responses = 732). The black line represents the cumulative percentage.

The problem rate was 17.4% (732 unfavorable responses out of total 4200 questions asked). Waiting time for appointment, waiting time on gastroscopy day and discomfort during procedure constituted over 90% of these unfavorable responses (Figure 2).

### Waiting time for appointment

**Figure 3:**
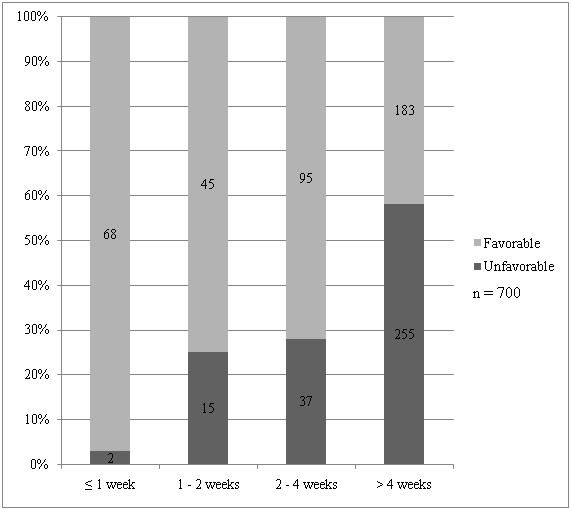
Patient responses towards waiting time for appointment across the different duration of waiting time.

Nearly two thirds of the patients had to wait for more than 4 weeks for their appointment (10% waited ≤ 1 week, 9% waited 1 – 2 weeks, 19% waited 2 – 4 weeks, 62% waited > 4 weeks). Favorable response diminished to undesirable level (from 79.4% to 41.8%) when waiting time for appointment exceeded 4 weeks (Figure 3). Patients with shorter waiting time for appointment (p < 0.001), those over 55 years old (p-value = 0.022) and those who never had a gastroscopy before (p < 0.001) were more likely to give favorable response towards waiting time for appointment on univariate analysis. Gender, ethnicity and education level did not affect patient satisfaction towards waiting time for appointment (data not shown). Only shorter waiting time for appointment (p < 0.001) and history of previous gastroscopy (p = 0.001) were independent predictors of favorable response towards waiting time for appointment on multivariate analysis.

### Waiting time on gastroscopy day

**Figure 4:**
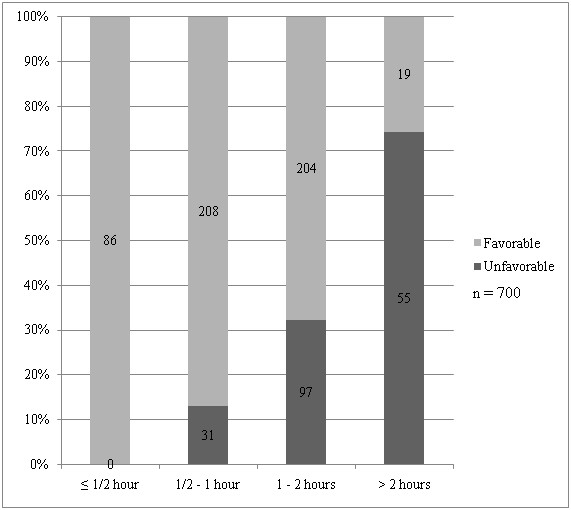
Patient responses towards waiting time at Endoscopy Suite across the different duration of waiting time.

More than half of the patients had to wait for over 1 hour for their turn on gastroscopy day (12% waited ≤ ½ hour, 34% waited ½ – 1 hour, 43% waited 1 – 2 hours, 11% waited > 2 hours). Favorable response diminished to undesirable level (from 90.5% to 67.8%) when waiting time on gastroscopy day exceeded 1 hour (Figure 4). Age, gender, ethnicity, education level and history of previous gastroscopy did not affect patient satisfaction towards waiting time on gastroscopy day (data not shown). Only shorter waiting time (p < 0.001) was found to be an independent predictor of favorable response towards waiting time on gastroscopy day on multivariate analysis.

### Discomfort during procedure

Twenty three percent of patients gave unfavorable response for comfort during procedure. Younger patients (55 years old or less) (p = 0.002), females (p = 0.007), and patients not given sedation (p < 0.001), given lower dosage of sedation (p < 0.001) or failed to achieve adequate (moderate) sedation according to MOAASS (p < 0.001) were more likely to give unfavorable response towards comfort level during procedure. However, only female gender and failure to achieve adequate (moderate) sedation according to MOAASS were independent predictors of unfavorable response towards comfort level during procedure.

### Factors associated with favorable overall rating

Ninety six percent of patients gave favorable response for overall rating. The following factors were associated with favorable overall rating on univariate analysis: use of sedation, achieving at least moderate level of sedation during procedure, favorable response to each of the six questions and waiting time gastroscopy day of one hour or less. Factors that were associated with favorable overall rating on multivariate analysis are favorable responses to the following: waiting time for appointment, waiting time on gastroscopy day, personal manner of endoscopist, explanation given by endoscopist and comfort level during procedure (Table 2). Majority of patients would return to the same physician (96.3%) and to the same centre (99.7%) should they need to undergo the same procedure in the future. Patients who gave favorable overall rating were more likely to do so (data not shown).

**Table 2:** Univariate and multivariate analysis of patient demographics, procedure-related information and response to questions Q1 to Q6 with patient overall rating

**Factors**	**Overall Satisfaction**		**Unadjusted OR**	**95% CI**	**p value**	**Adjusted OR**	**95% CI**	**p value**
	**Favorable**	**Unfavorable**						
Age								
≤ 55	312 (96.3%)	12 (3.7%)	0.81	0.38, 1.73	0.589	-	-	-
> 55	359 (95.5%)	17 (4.5%)						
Sex								
Male	314 (96.3%)	12 (3.7%)	0.80	>0.38, 1.71	0.568	-	-	-
Female	357 (95.5%)	17 (4.5%)						
Ethnicity								
Malay	199 (98%)	4 (2%)						
Chinese	276 (95.2%)	14 (4.%)	0.69	0.43, 1.10	0.120	0.61	0.31, 1.19	0.149
Indian	188 (94.5%)	11 (5.5%)						
Others	8 (100%)	0 (0%)						
Education Level								
Primary	110 (95.7%)	5 (4.3%)						
Secondary	287 (96%)	12 (4.0%)	0.98	0.66, 1.46	0.915	-	-	-
Tertiary	173 (96.1%)	7 (3.9%)						
None	101 (95.3%)	5 (4.7%)						
Previous gastroscopy								
Yes	286 (95.7%)	13 (4.3%)	1.09	0.52, 2.31	0.814	-	-	-
No	385 (96%)	16 (4%)						
Duration of gastroscopy								
≤ 10 minutes	392 (96.3%)	15 (3.7%)	0.76	0.36, 1.61	0.474	-	-	-
> 10 minutes	279 (95.2%)	14 (4.8%)				-	-	-
Sedation								
Yes	618 (96.4%)	23 (3.6%)	0.33	0.13, 0.84	0.015	0.51	0.12, 2.19	0.368
No	53 (89.8%)	6 (10.2%)						
Midazolam dose								
≤ 2.5 mg	228 (95.8%)	10 (4.2%)	1.32	0.57, 3.05	0.521	-	-	-
> 2.5 mg	390 (96.8%)	13 (3.2%)						
OAASS								
2 – 4 (moderately sedated)	584 (97.7%)	14 (2.3%)	0.14	0.07, 0.30	<0.001	0.65	0.18, 2.32	0.508
5 (minimally sedated)	87 (85.3%)	15 (14.7%)						
Response for waiting time for appointment								
Favorable	386 (98.7%)	5 (1.3%)	6.50	2.32, 19.67	<0.001	3.73	1.18, 11.9	0.026
Unfavorable	285 (92.2%)	24 (7.8%)						
Waiting time for appointment								
≤ 4 weeks	251 (95.8%)	11 (4.2%)	1.02	0.48, 2.20	0.954	-	-	-
> 4 weeks	420 (95.9%)	18 (4.1%)						
Response for waiting time at Endoscopy Suite								
Favorable	510 (98.6%)	7 (1.4%)	9.96	3.95, 26.14	<0.001	6.23	1.98, 19.63	0.002
Unfavorable	161 (88%)	22 (12%)						
Waiting time at Endoscopy Suite								
≤ 1 hour	320 (98.5%)	5 (1.5%)	0.23	0.09, 0.61	0.001	0.63	0.18, 2.18	0.465
> 1 hour	351 (93.6%)	24 (6.4%)						
Response to personal manner of endoscopist								
Favorable	657 (97.5%)	17 (2.5%)	33.13	12.2, 90.69	<0.001	15.15	3.73, 61.58	0.000
Unfavorable	14 (53.8%)	12 (46.2%)						
Response to personal manner of staff/nurses								
Favorable	660 (96.9%)	21 (3.1%)	22.86	7.46, 69.81	<0.001	1.29	0.21, 7.82	0.784
Unfavorable	11 (57.9%)	8 (42.1%)						
Response to explanation given								
Favorable	646 (97%)	20 (3%)	11.36	4.39, 30.38	<0.001	7.40	2.01, 27.32	0.003
Unfavorable	25 (73.5%)	9 (26.5%)						
Response to comfort level during endoscopy								
Favorable	532 (98.7%)	7 (1.3%)	12.03	4.76, 31.66	<0.001	5.41	1.73, 16.90	0.004
Unfavorable	139 (86.3%)	22 (13.7%)						

OAASS = observer assessment for alertness/sedation scale.

### On-site survey and phone-back survey

**Figure 5:**
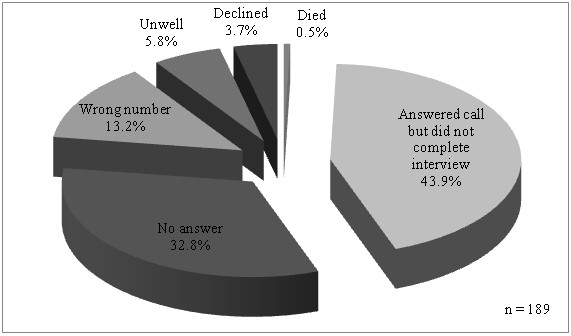
Reasons for unsuccessful phone-back interview.

Mean interval of phone-back interview from procedure day was 12 ± 6 days. Of the 700 patients interviewed on-site, only 511 patients (73%) completed the phone-back interview. The reasons for unsuccessful phone-back interview are shown in Figure 5. Patients aged more than 55 years old were more likely to complete the phone-back interview than patients less than 55 years old (p = 0.033). Response to phone-back interview was not influenced by gender or race (data not shown).

The mean total score for Q1 to Q7 was 23.0 ± 3.8 for on-site interview and 22.9 ± 3.5 for phone-back interview. The median total score was 22 and was the same for both groups. Because the Likert scale that we used only had 5 possible values, we often ended with identical median score for the on-site and phone-back groups. The mean scores for waiting time for appointment, waiting time on gastroscopy day, personal manner of endoscopist and comfort during procedure were higher while the mean scores for personal manner of nurse/staff and explanation given were lower in the phone-back group. The differences were statistically significant for waiting time for appointment, personal manner of nurse/staff and explanation given (Table 3). The mean scores were same for overall rating for both groups.

**Table 3:** Comparison of mean and median scores for questions Q1 to Q7 for on-site interview and phone interview

**Questions**	**Phone-back**			**On-site**			**p value**
	**Mean (SD)**	**Median (25Q – 75Q)**	**Low Score Details**	**Mean (SD)**	**Median (25Q – 75Q)**	**Low Score Details**	
Waiting time for appointment	2.89 (0.69)	3 (2 – 3)	2 (141), 1 (0)	2.78 (0.92)	3 (2 – 3)	2 (199), 1 (18 )	0.001
			< 2(141or 27.6%)			< 2(217 or 42.5%)	
Waiting time at Endoscopy Suite	3.07 (0.71)	3 (3 – 3)	2 (97), 1 (0)	3.06 (0.85)	3 (3 – 4)	2 (99), 1 (14)	0.868
			< 2(97 or 19%)			< 2(113 or 22.1%)	
Personal manner of endoscopist	3.60 (0.73)	4 (3 – 4)	2 (13), 1 (2)	3.57 (0.74)	4 (3 – 4)	2 (19 ), 1 (1)	0.329
			< 2(15 or 2.9%)			< 2(20 or 3.9%)	
Personal manner of nurses/staff	3.32 (0.71)	3 (3 – 4)	2 (39), 1 (4)	3.53 (0.68)	3 (3 – 4)	2 (13), 1 (0)	<0.001
			< 2(43 or 8.4%)			< 2(13 or 2.5%)	
Explanation given	3.36 (0.74)	3 (3 – 4)	2 (36), 1 (4)	3.43 (0.73)	3 (3 – 4)	2 (21), 1 (1)	0.035
			< 2(40 or 7.8%)			< 2(22 or 4.3%)	
Comfort level during procedure	3.22 (0.85)	3 (3 – 4)	2 (54), 1 (19)	3.00 (0.98)	3 (3 – 4)	2 (91), 1 (22)	0.288
			< 2(73 or 14.3%)			< 2(113 or 22.1%)	
Overall satisfaction	3.47 (0.72)	3 (3 – 4)	2 (24), 1 (2)	3.47 (0.70)	3 (3 – 4)	2 (16), 1 (0)	0.371
			< 2(26 or 5.1%)			< 2(16 or 3.1)	

## Discussion

Evaluation of patient satisfaction and addressing areas of dissatisfaction is an important aspect of healthcare services and is a measure of quality of service provided. This process has been found to be useful in improving standards of endoscopy centers including performance of endoscopists, and possibly the reputation of endoscopy centers in the long run [2]. Patient satisfaction also affects perception of the population at large towards endoscopic services and can have significant impact on patient willingness to undergo endoscopic procedures regardless of whether the patient has had endoscopy before.

Different questionnaires have been used to assess patient satisfaction towards gastrointestinal endoscopy [10,14,15]. The American Society of Gastrointestinal Endoscopists (ASGE) recommended the use of the mGHAA-9 questionnaire to measure patient satisfaction [15]. However, mGHAA-9 does not contain a question on patient comfort which has been found to be an important factor influencing patient satisfaction [16]. It was also noted that patients had difficulty answering the question on technical skills of endoscopist found in mGHAA-9 [14]. We anticipated a similar problem with our patients and have substituted this question with one on patient comfort.

As different health care system may vary in term of aspects that patients consider being important [17], areas of dissatisfaction unique to local patient population should be identified and analyzed and corrective measures instituted for improvement accordingly. Five independent factors affecting overall rating were identified in our population: waiting time for appointment, waiting time on gastroscopy day, personal manner of physician, adequacy of explanation and discomfort during procedure. Of these, waiting times and discomfort during procedure ranked the highest in terms of unfavorable responses.

Increasing number of patients scheduled for gastroscopy and limited resources have resulted in long appointment waiting times in our center while prolonged waiting on the day of gastroscopy may be the result of combination of factors including over-scheduling of cases for each session. Nearly half of our patients were dissatisfied with waiting time for gastroscopy appointment while close to one quarter were unhappy with their waiting on gastroscopy day. As dissatisfaction towards appointment waiting time could have resulted in a proportion of patients transferring to another outpatient gastroscopy service, our figure could be an under-estimation of the true proportion of patients who were dissatisfied in this aspect. Waiting times for endoscopy appointment and on endoscopy day are problems not restricted to our center but appear to be major causes of unfavorable responses in other centers as well [18-21]. In this aspect, it is vital that increasing patient load is matched by increasing allocation of resources to maintain a service that meets the expectations of not only patients but also of healthcare providers.

Discomfort during procedure was recognized as the main cause of patient dissatisfaction in some studies [22,23]. Despite using proven measures to minimize discomfort during gastroscopy, including pharyngeal anesthesia and conscious sedation [7-9,24], nearly a quarter of our patients were not satisfied. We found that patients who were only minimally sedated were more likely to give unfavorable response for comfort during procedure (data not shown). In this aspect, routine use of OAASS as an objective measure of adequate (moderate) sedation prior to commencing the procedure may be of benefit. Besides sedation, other factors such as the diameter of the endoscope [25] and level of experience of the endoscopist [23] may affect the level of comfort during the procedure. However, our study was not designed to look into these factors.

Besides waiting times and discomfort during procedure, other factors have yielded unfavorable responses from our patients. However, utilizing the principle of “vital few and trivial many” [26], we identified that waiting times and discomfort during procedure constituted to nearly 90% of the problems faced by our patients. By focusing on improvement in these aspects, there is great likelihood of substantially reducing the problem rate among patients attending our outpatient gastroscopy service. Based on our analysis, aiming for gastroscopy appointment waiting time of within 1 month and waiting time on gastroscopy day of within 1 hour will result in an improved rate of favorable response to nearly 80% and over 90%, respectively. However, as this is a single-center study, this result may not be generalizable to other populations. Nevertheless, by using a similar approach, other centers may be able to gauge the waiting times that are acceptable for their patient population.

Previous studies have shown that survey collection method may impact on subject responses. Phone-back methods are generally associated with more favorable responses compared to mail-back methods [27-30] although some studies did not find any difference between the two methods [11,31,32]. Among patients who underwent endoscopy, satisfaction scores were better when surveys were completed on-site compared with when they were mailed back [10,22]. Interesting terms such as “social desirability response” bias (patients giving better responses than they feel because they feel it is more acceptable) and “ingratiating response” bias (patients giving better responses than they feel because they wish to ingratiate themselves with their providers) have been used for the phenomenon where satisfaction scores were better when obtained through more personal and earlier communications with patients [6]. Success rates are also generally better with on-site and phone-back methods compared with mail-back methods [11,27,28,32]. To our best knowledge, no studies have been conducted to compare on-site interview versus phone-back interview in evaluation of patient satisfaction of endoscopy services.

We found that satisfaction scores were better for waiting time for appointment but lower for personal manner of nurses/staff and for adequacy of explanation during phone-back interview compared with on-site interview. We hypothesize that dissatisfaction towards waiting time for appointment naturally diminished over time after the procedure helped in reassuring patients when there was nothing wrong or facilitated effective treatment following accurate diagnosis of the underlying condition. On the other hand, patients may have been more reluctant to give a low score for personal manner of nurses/staff and for adequacy of explanation during the on-site interview while still within the vicinity of the Endoscopy Suite. There was no significant difference in satisfaction scores for other questions, including overall rating between the two methods although there was a trend towards better scores during phone-back interview. This factor should be considered when comparing individual items of the questionnaire between centers or between two time-points in the same center if different methods (i.e. on-site vs. phone-back) were used. However, overall rating and some of the items may still be comparable. Caution should also be exercised when interviewing patients through phone-back when they have missed their on-site interview in patient satisfaction studies as some of the results may not be comparable when obtained using the two different methods. We would prefer on-site interview when conducting satisfaction survey due to its better success rate and because it is arguably easier and less costly to administer compared with phone-back interview.

Our center practices an open-access outpatient gastroscopy service receiving patients from primary care clinics, other specialist clinics and those discharged from in-patient wards in addition to patients from the gastroenterology clinic. Hence data from this study is generalizable to populations scheduled for gastroscopy at large. Despite our efforts, this study has several limitations. Firstly, we conducted the interview on the same group of patients and the response to the second interview may be biased by that of the first interview. Our findings should be confirmed with randomized study of two distinct groups of patients i.e. one for on site interview while the other for phone interview. Secondly, while the questionnaire used has obvious face validity, it has not been formally validated for our local population. There is currently no formally validated satisfaction survey questionnaire for endoscopy for our local population.

## Conclusion

We found waiting times and discomfort during procedure to be the main causes for patient dissatisfaction towards the outpatient gastroscopy service of an Asian tertiary hospital. Measures to reduce waiting times for gastroscopy appointment and on gastroscopy day to less than 1 month and 1 hour, respectively may improve patient satisfaction substantially. Phone-back interview may result in different patient satisfaction scores compared with on-site interview. This should be taken into account when comparing results obtained using these two different methods. On site interview may be preferred as it gives a better success rate and is arguably easier and less costly to administer.

## Competing interests

The authors declare that they have no competing interests.

## Authors’ contributions

NA contributed through data collection, data entry, data analysis and interpretation, and drafting of the article. WKC contributed through conception and design, data analysis and interpretation, and critical revision of the article for important intellectual content. KLG contributed through conception and design, critical revision of the article for important intellectual content, and final approval of the article. All authors read and approved the final manuscript.

## Pre-publication history

The pre-publication history for this paper can be accessed here:

http://www.biomedcentral.com/1471-230X/12/96/prepub
